# Theatre etiquette Delphi: the development of a guide on professional conduct and best practices in operating environments

**DOI:** 10.1308/rcsann.2025.0040

**Published:** 2025-06-17

**Authors:** M El Boghdady, J Hardie, PA Brennan, TED Collaborative Group

**Affiliations:** ^1^ St George’s University Hospitals NHS Foundation Trust, UK; ^2^ University Hospital Southampton, UK; ^3^ Portsmouth Hospitals University NHS Trust, UK

**Keywords:** Operating theatre, Respect, Etiquette, Behaviour

## Abstract

**Introduction:**

The importance of non-technical skills (NTS) to surgical performance and patient safety has been increasingly recognised by surgical teams. Inductions for new surgical team members in theatre often provide insufficient, non-standard and ‘ad hoc’ training in theatre behaviour and etiquette. We conducted a Delphi consensus study among senior surgeons to develop standardised guidance on theatre etiquette for those unfamiliar with the theatre environment, including resident surgical trainees and medical students.

**Methods:**

An international Delphi process of two rounds was conducted. An electronic survey was distributed among senior surgeons, anaesthetists and senior scrub nurses/practitioners, with participants recruited via surgical societies. Participants were asked to rank each statement on a Likert scale of 1 to 5. Consensus was considered if achieved for any statement for which 75% or more indicated agreement. The study was registered with the Open Science Framework.

**Results:**

A total of 261 participants completed the Delphi process; 239 valid responses were included in round 1, with a 23% dropout in round 2. Participants were from 23 countries, 66% were from the UK, 58.2% were male, 51% were from the 30 to 40-year age group, 39% were consultant surgeons and 49% were senior trainees. General surgeons made up 68.6% of respondents, trauma and orthopaedic surgeons 13.4%, healthcare practitioners 2.1% and anaesthetists 1.3%. Thirteen statements were excluded, and 29 reached agreement and were included in the final guidance.

**Conclusion:**

There was agreement among a large international group of surgeons to develop a standardised guidance for theatre etiquette, addressing most of the key aspects of professional conduct and team dynamics. We anticipate that this guidance will serve as a valuable resource for orienting new members of the surgical team, providing a clear framework for maintaining professionalism and fostering effective communication within the theatre environment.

## Introduction

The importance of non-technical skills (NTS) to enhance surgical performance has been increasingly recognised. These include effective teamwork, communication, decision-making and situation awareness within the operating theatre. This recognition has led to the increased integration of NTS training into both undergraduate and postgraduate medical education programmes.^1,2^ The British General Medical Council explicitly highlights NTS as a fundamental component of Good Medical Practice, emphasising their significance in delivering safe and effective care. Similarly, the Intercollegiate Surgical Curriculum Programme has incorporated non-technical competencies into its framework, embedding them within workplace-based assessments.

Inductions for new theatre team members including medical students in surgical placements or resident trainees with limited theatre experience often provide insufficient, non-standard and ‘ad hoc’ training in theatre behaviour and etiquette. This lack of structured and comprehensive onboarding can leave new theatre team members unprepared for the unique operating theatre environment. As a result, they may inadvertently breach professional conduct, leading to moments of embarrassment or discomfort. More importantly, inadequate training can undermine team communication, increasing the risk of errors that may compromise patient safety and overall operational efficiency in the operating theatre.

Although previous advice and tips on theatre etiquette have been offered, this has been limited to preparing the surgical safety checklist and performing the surgical scrub.^3,4^ To date, there are no publications or references in the literature to outline comprehensive principles of theatre and operating table etiquette. Recognising that being an effective surgeon involves more than just being a good ‘pair of hands’, we aimed to conduct a Delphi consensus among senior surgeons to develop standardised guidance on theatre etiquette for those unfamiliar with the operating theatre environment, including resident surgical trainees and medical students.

## Methods

An international Delphi process in form of two rounds was undertaken for a period of 4 months. A Delphi consensus is a well-established technique by which iterative anonymous opinion is given within a group of participants to define agreement on a series of statements with the avoidance of participant bias.^5^ The questions were formulated and developed among the research group members. The questions were formulated by our research group, which collaboratively developed the survey based on the study objectives. The initial set of questions was then reviewed by senior members with expertise in the field to ensure clarity, relevance, and validity. Following this, the survey was piloted among a small group of participants to assess comprehension and refine any ambiguous or unclear items before finalising the questionnaire.

Two Delphi rounds were circulated via electronic survey among senior surgeons (consultants, fellows or senior trainees), anaesthetists (consultants, fellows or senior trainees) and senior scrub nurses/practitioners. Participants were recruited by email invitation and social media posts from surgical societies. Two follow-up emails were sent to non-respondents. The power analysis of the study was conducted to ensure the study was adequately powered to analyse consensus between rounds.

Participants were asked to answer questions relating to demographic data, including age, gender, level of training and specialty. In addition, they were asked to generate statements addressing preoperative, intraoperative and postoperative related topics. In round 1, half of the statements were negatively worded to decrease the risk of bias. Participants were invited to contribute additional statements, which were subsequently incorporated in round 2. Participants were asked to rank each statement on a Likert scale of 1 (strongly disagree) to 5 (strongly agree). Participation was voluntary, and participants were asked whether they would like to participate in the next round of the Delphi, and whether they preferred to disclose their details or remain anonymous.

The study considered that consensus was achieved for any statement for which 75% or more respondents indicated agreement (Likert scale 4 or 5). The statements that did not reach agreement in round 1 were excluded in round 2 (Appendix 2 – available online). Analysing authors were blind to participant roles and demographics. Invitations to participate in round 2 were sent to the email addresses of the participants who had indicated in previous round that they wished to continue to participate. Data were analysed using Microsoft Excel (Microsoft Corp, Redmond, WA). The study was registered with the Open Science Framework.

## Results

A total of 261 participants completed the Delphi process and 239 valid responses were included. Based on the medium effect size (*d* = 0.5) and a significance level of α = 0.05, a target of 180 participants in round 2 was planned to achieve a power of 0.99. Despite a dropout rate of approximately 23% between rounds, the final round 2 sample size matched the target, ensuring a power of 1.0. In round 1 of the Delphi process, 30 statements were generated and presented to participants. Statements that did not achieve consensus were excluded after this round. In addition, participants were invited to suggest new statements in the preoperative, intraoperative or postoperative categories. Based on their input, three new statements were added, resulting in a total of 33 statements in round 2. Thirteen statements were excluded, and 29 reached agreement and were included in the final guidance.

### Participant demographics

Of the participants, 58.2% were male and 41.8% were female. Fifty-one per cent were aged 30–40 years, 21.8% were aged 41–50, 9.6% were aged 51 to 60 and 2.5% were older than 60.

### Geographic representation

**Figure 1:**
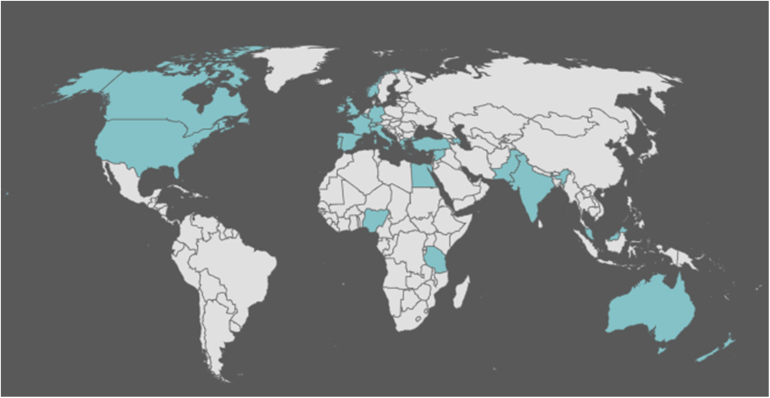
Worldwide distribution (blue) of participants who completed the Delphi process

Sixty-six per cent of participants were from the United Kingdom (UK); other representation included participants from the United States, Australia, Canada, Republic of Ireland, Belgium, Germany, Spain, Italy, Turkey, Syria, Egypt, Azerbaijan, Tanzania, Malaysia, Greece, Norway, India, Pakistan, Portugal, Nigeria, Switzerland and France (Figure 1).

### Level of training representation

Some 39.7% of participants were consultant surgeons, 49% were senior trainees, including specialist registrars and fellows, and the remainder were from anaesthesia and healthcare professions.

### Profession and specialty representation

Of the participants, 68.6% were general surgeons, 13.4% were from trauma and orthopaedics, 2.1% were healthcare practitioners, 3.3% were anaesthetists, and 12.6% were from other surgical subspecialties such as cardiothoracic surgery, otolaryngology, neurosurgery, oral and maxillofacial surgery, paediatric surgery, plastic surgery, vascular, transplant and breast surgery.

**Figure 2:**
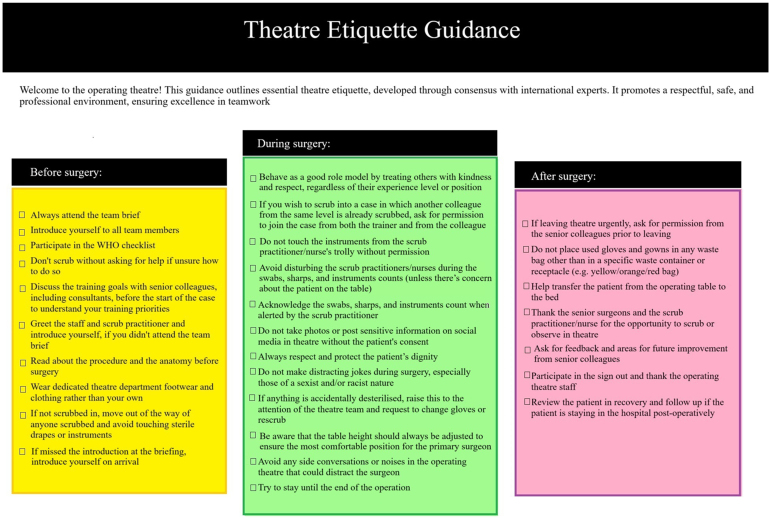
Theatre etiquette guidance

Analysis of the preoperative, intraoperative and postoperative-related topics was performed in round 1 and round 2 (Table 1). Based on this Delphi study, new theatre etiquette guidance was developed (Figure 2).

**Table 1:** Consensus recommendations in both Delphi rounds

	**Round 1 agreement (%)**	**Round 2 agreement (%)**
**Preoperative-related topics**		
Someone who is unfamiliar with the operating theatre environment (student or resident trainee) should:		
Always attend the team brief	94.2	90.3
Introduce themselves to all team members	93.3	93.7
Participate in the WHO checklist	85.7	85.6
Don’t scrub without asking for help if unsure how to do so	90	89.8
Discuss the training goals with senior colleagues, including consultants, before the start of the case to understand their training priorities	92.9	92.2
Greet the staff and scrub practitioner and introduce themselves, if they didn’t attend the team brief	90.8	89.8
Should read about the procedure and the anatomy before surgery		94.7
They should wear dedicated theatre department footwear and clothing rather than their own		86.4
If not scrubbed in, they should move out of the way of anyone scrubbed and avoid touching sterile drapes or instruments		98.2
If missed the introduction at the briefing, they should be introduced or introduce themselves on arrival		97.6
**Intraoperative-related topics**		
Should behave as a good role model by treating others with kindness and respect, regardless of their experience level or position	99.5	100
If they wish to scrub into a case in which another colleague of a similar grade is already scrubbed, it is incumbent to ask for permission to join the case from both the trainer and from the colleague	88.7	94.7
It is not acceptable to touch the instruments from the scrub practitioner/nurse’s trolly without permission	76.1	76.5
Avoid disturbing the practitioners/nurses during the swabs, sharps, and instruments counts (unless there’s concern about the patient on the table)	90.4	100
Acknowledge the swabs, sharps, and instruments count when alerted by the scrub practitioner	98	99.4
It is not acceptable to take photos or post sensitive information on social media in theatre without the patient's consent	94.1	99.4
Should always respect and protect the patient’s dignity	98.7	99.4
It is not acceptable to make distracting jokes during surgery, especially those of a sexist and/or racist nature	94.2	91.7
If anything is accidentally desterilised, it is incumbent to raise this to the attention of the theatre team and request to change gloves or rescrub	97.5	99.4
Should be aware that the table height should always be set to be the most comfortable position for the primary surgeon	78.2	78.1
They should avoid any side conversations or noises in the operating theatre that could distract the surgeon		85.2
Should try to stay until the end of the operation		77
**Postoperative-related topics**		
If leaving theatre urgently, it is incumbent to ask for permission from the senior colleagues prior to leaving	86.2	87.6
It is not acceptable to place used gloves and gowns in any waste bag other than in a specific waste container or receptacle (e.g. yellow/orange/red bag)	86.2	97.6
Help transfer the patient from the operating table to the bed	83.6	92.9
Should thank the senior surgeons and the scrub practitioner/nurse for the opportunity to scrub or observe in theatre	75	85.2
Ask for feedback and areas for future improvement from senior colleagues	95.4	97.6
Should participate in the sign out and thank the operating theatre staff		86.4
Should review the patient in recovery and follow up if the patient is staying in the hospital postoperatively		84

## Discussion

NTS have a crucial role in improving team performance, and improving patient safety.^6^ Effective communication and understanding of human behaviour and high levels of situation awareness can contribute to minimising errors. Recognising the impact of stress, fatigue and hierarchy on interactions enables teams to implement strategies that foster open dialogue and mutual respect, empowering all members to voice their concerns or observations without fear.^6,7^

The culture of surgery is unique, and early-career surgeons may come from diverse backgrounds and levels of experience.^8^ Senior trainees are expected to have a thorough understanding of operating theatre etiquette, gained through their experience and exposure to practice. For example, they may introduce themselves to patients, attend the consenting process and discuss their educational aims for the day with trainers. Conversely, resident trainees who are starting their careers may not yet possess this knowledge or awareness of theatre etiquette. As a result, it is essential to provide those less familiar with the operating environment with clear and consistent guidance to help them navigate expectations and develop their communication skills effectively.^9^

Operating theatre etiquette is a distinctive and integral aspect of the surgical profession, reflecting its unique culture and values. This etiquette, crucial to maintaining professionalism and a harmonious working environment, has historically been learned informally by senior surgeons and staff members working as role models for the new generations of surgeons. We found that operating theatre etiquette is similar internationally, despite geographical and local cultural differences. Ensuring this tradition is upheld and passed on to new generations is essential, not only to preserve the ethical and professional nature of the field, but also to adapt and modernise practices, while moving away from outdated hierarchical traditions.

Some statements in the questionnaire were highlighted by participants, such as whether surgeons should wear their own clothes rather than hospital-provided workwear. The statement was intended to be exclusively about scrubs and footwear, rather than directed to any faith or religious groups. Free-text comments, however, drew attention to the fact that surgeons could wear hijabs for religious purposes if this complies with infection control and cleanliness guidelines. Uniforms and Workwear Guidance for NHS Employers, published in April 2020, promoted examples of good practice related to hijabs in theatres, serving the three objectives of patient safety, public confidence and staff comfort.^10^

Although this study was primarily intended to create a guide for medical staff and students, participants highlighted points relevant to the wider team. For instance, scrub practitioners should ask the surgeon whether it is appropriate to start the surgical count of swabs and instruments to avoid disrupting critical steps in the operation, or causing distraction. Participants emphasised that that the instruments trolly should not be referred to as the nurses’ or practitioners’ table, and it may be acceptable in some critical, unexpected situations to touch and use the instruments without prior permission.

Maintaining a professional and respectful atmosphere in the operating theatre is essential for both the safety of patients and the well-being of the surgical team. Although a relaxed environment with a cohesive team can make the surgical profession more appealing, comments and jokes of a sexist or racist nature are inappropriate and unprofessional. Such behaviour not only risks distracting the team from their primary task and can affect the patient safety, but also fosters a toxic environment that can threaten and discomfort students or resident surgeons.^8,11^ These behaviours are indicative of poor judgement and undermine the inclusivity and respect that should characterise any workplace, particularly one as critical as a surgical theatre.

Another point was highlighted regarding the appropriateness of requiring resident surgeons and students to assist with postoperative tidying in the theatre, balancing this expectation with their demanding schedules and educational priorities. Although offering to help the staff post-surgery fosters teamwork and respect within the theatre, many participants emphasised that making this a mandatory requirement might detract from resident surgeons’ limited time to focus on learning and completing essential tasks. Instead, an alternative approach could be to encourage such gestures as voluntary acts of collegiality rather than essential and expected duties, allowing trainees to prioritise their core learning responsibilities. This statement did not receive consensus during round 2 of the Delphi, highlighting the importance of maintaining a balance between fostering team dynamics and respecting the workload of resident surgeons.

### Study limitations

The Delphi method, although valuable for gaining consensus among experts, has inherent limitations, particularly in the context of a study with two rounds targeting senior surgeons, anaesthetists and senior scrub practitioners with busy schedules. One limitation of this study was the potential for selection bias, because participants self-select in response to email invitations and social media posts, potentially skewing the sample towards those with strong opinions or greater digital accessibility. The international aim of the study may also face challenges owing to varying levels of engagement across regions, influenced by cultural differences, language barriers or time constraints of operating theatre team members. However, it is anticipated that the risk of these biases has been reduced with a sample size of more than 200 participants from senior surgeons and operating theatre staff members from the UK and internationally. The demographic data also reflected gender diversity in participants from different specialties and various levels of training.

## Conclusion

There was an agreement among a large international group of surgeons to develop a standardised guidance for theatre etiquette, addressing most of the key aspects of professional conduct and team dynamics. We anticipate that this guidance will serve as a valuable resource for orienting new members of the surgical team, providing a clear framework for maintaining professionalism and fostering effective communication within the theatre environment. It is anticipated that through use of the guidance, effective communication among team members and the new joiners will be enhanced, ultimately improving the teaching and work environment, as well as prioritising patient safety and ensuring a high standard of care.
